# Supplementary material for: Daily Aspirin Reduced the Incidence of Hepatocellular Carcinoma and Overall Mortality in Patients with Cirrhosis
Source: Cancers (Basel). 2023 May 27;15(11):2946. doi: 10.3390/cancers15112946 (PMC10251874; doi:10.3390/cancers15112946)
Supplement: Supplementary file 1 [file cancers-15-02946-s001.zip › cancers-2353875-supplementary.pdf]

Online Supplementary Material

Daily Aspirin Reduced the Incidence of Hepatocellular Carcinoma and Overall Mortality in Patients with Cirrhosis

Chern-Horng Lee, MD;[1] Chiu-Yi Hsu, MS;[2] Tzung-Hai Yen, MD, PhD;[3] Tsung-Han Wu, MD;[4] Ming-Chin Yu, MD, PhD;[4] Sen-Yung Hsieh, MD, PhD[5,6*]

[1]Department of General Medicine, Chang Gung Memorial Hospital, Linkou, Taoyuan, Taiwan

[2]Center for Big Data Analytics and Statistics, Chang Gung Memorial Hospital, Linkou, Taoyuan, Taiwan

[3]Department of Nephrology, Chang Gung Memorial Hospital, Linkou, Taoyuan, Taiwan

[4]Department of General Surgery, Chang Gung Memorial Hospital, Linkou, Taoyuan, Taiwan

[5]Department of Gastroenterology and Hepatology, Chang Gung Memorial Hospital, Linkou, Taoyuan, Taiwan

[6]College of Medicine, Chang Gung University, Taoyuan, Taiwan

Correspondence: Dr. Sen-Yung Hsieh, Department of Gastroenterology and Hepatology, Chang Gung Memorial Hospital, Linkou, Taoyuan 333, Taiwan; Phone: +886-0975368031; Fax: 886-3-3272236; E-mail: siming@cgmh.org.tw; siming.shia@msa.hinet.net

Supplementary Methods S1. The ICD coded used in this study

	Comorbidity disease	ICD-9 code	ICD-10 code
1	Cardiovascular disease (CVD)		
	Angina pectoris,	4139, 413.9	I20
	Acute myocardial infarction	410, 410.9,	I21.9
	Athersclerosis	4409,	I25.1
	Coronary arterial disease (CAD)	4140,	I25.10
	Old myocardial infarction	412,	I25.2
	Other acute ischemic heart disease		I24
	Ischemia heart disease (IHD)	414.9,	I25
	Certain current complications following ST elevation (STEMI) and non-ST elevation (NSTEMI) myocardial infarction (within the 28 days period)		I22, I23
2	Cerebral vascular accident (CVA)		
	Transient ischemia attack (TIA)	435, 4359,	G45.9, G45
	Cerebral Infarction	4349,	I63
	Stroke	433–436, 436, 4370,	G46
	Sequelae of Stroke	438.9,	I69.3
3	Diabetes mellitus (DM)	2500, 25001, 25050, 25060, 357.2, 25080, 250, 25070, 250.7, 785.4,	E11, E10
4	Fatty liver	5718,	k70.0, k76.0
5	Obesity	2780,	E66
6	Cirrhosis	5712, 5715, 5716,	K702, K7030, K7031, K74, k74.60, k70.3
7	Biliary cirrhosis		K74.5
8	HBsAg	7030,	B16
9	Anti-HCV antibody	7051,	B18
10	Hepatitis	57140, 5710, 5711,	K73 K70.1 B17
11	Hepatocellular carcinoma	155.0	C22.0
12	Gastrointestinal bleeding	578.9, 578	K92, K92.2 K25-K28, K29. K57. K31.811

Covariates were defined using ICD classifications.

Supplementary Methods S2. Definitions of Exposures and Covariates

Entity	Source	ATC
Anti-Platelet agent		
Aspirin	Prescribed Drug Register	B01AC06
Aggrenox (Dipyridamole + Aspirin)		B01AC30
Clopidogrel		B01AC04
Dipyridamole		B01AC07
Ticagrelor		B01AC24
Iloprost		B01AC11
Tirofiban		B01AC17

Abbreviations: ATC, Anatomical Therapeutic Chemical classification system;

Supplementary Table S1. Clinical characteristics of cirrhotic patient with and without aspirin use (Study A)[a]

Characteristics	Untreated (n = 2270)		Treated (n = 1135)		*p* - Value[b]	SMD	
	n	%	n	%			
Male (n, %)	1493	65.77	754	66.43	0.7012	0.01	chi-square
Age (y, mean±SD)	60·39	13.85	61.16	12.83	0.1057	0.00	t test
Age >65 y	873	38.46	433	38.15	0.8615	0.01	chi-square
Aspirin (days)							
Mean (±SD)			861.09	875.83			
Median (IQR)			524	(201-1240)			
Comorbidities (n, %)							
Cardiovascular diseases	257	11.32	129	11.37	0.9695	0.00	chi-square
Cerebral vascular diseases	304	13.39	149	13.13	0.8305	0.01	chi-square
Diabetes	1006	44.32	499	43.96	0.8452	0.01	chi-square
Fatty liver/ Obesity	93	4.1	51	4.49	0.5879	0.02	chi-square
Medications use (n, %)							
Interferons or nucleosides	399	17.58	223	19.65	0.1405	0.05	chi-square
Metformin	760	33.48	377	33.22	0.8775	0.01	chi-square
NSAID	1376	60.62	908	80	<0.0001	0.43	chi-square
Statin	683	30.09	345	30.4	0.8534	0.01	chi-square
PPI	1285	56.61	736	64.85	<0.0001	0.17	chi-square
H2 blocker	973	42.86	678	59.74	<0.0001	0.34	chi-square
Laboratory data (median, Q1 to Q3)							
AFP [ln(ng/mL); median (IQR)]	1.6	(1.13-2.22)	1.5	(1.04-2.06)	0.0823	-	Mann-Whitney
ALT ≥ 70 IU/L	429	20.57	242	21.67	0.4622	0.03	chi-square
Platelets ≥ 140 x 1000/ul	866	4.04	548	49.32	<0.0001	1.19	chi-square
Total Bilirubin > 3.0 mg/dl	335	16.59	103	9.44	<0.0001	0.21	chi-square
INR >1.5	257	13.69	71	6.85	<0.0001	0.23	chi-square
HBsAg (+)	524	33.44	235	27.65	0.0034	0.13	chi-square
HCV antibody (+)	592	33.24	372	38.19	0.0092	0.10	chi-square
MELD Score (mean±SD)	10·11	3.18	11.3	6.62	0.0032		t test
MELD Score	n	%	n	%	0.0037		chi-square
0 ~ 10	1977	87.09	950	83.7		0.10	
11 ~ 20	135	5.95	102	8.99		0.12	
21 ~ 30	158	6.96	83	7.31		0.01	
> 30	0	0	0	0		-	

[a]Cases selected by randomly 1:2 PSM by sex, age, CVD, CVA, DM, metformin, statin.

[b]All statistical tests were 2 tailed and used a type I error rate of 0.05 (p).

[c]AFP: alpha-fetoprotein; ALT: alanine aminotransferase; CI: confidence interval, CVD: cardiovascular disease; CVA: cerebrovascular attack; DM: diabetes mellitus; HBV: HBsAg positive; HCV: anti-HCV antibody; HR ： hazard ratio; INR: international normalized ratio; NSAID: nonsteroidal

anti-inflammatory drug; PPI: proton pumping inhibitor; PSM: propensity score matching; SMD: standardized mean difference.

Supplementary Table S2. Clinical characteristics of cirrhotic patients with vs without aspirin use (Study B: Laboratory data included for PSM)[a]

	Propensity Matched (1:2)					
**Characteristics**	**Untreated (N=1538)**		**Treated (N=769)**		**SMD**	***p* - Value[b]**
Male (n, %)	1022	66.45	524	68.14	0.04	0.4156
Age (mean±SD)	59.94	13.84	60.72	12.83	0.00	0.1808
Age >65	580	37.71	289	37.58	0.00	0.9515
Aspirin therapy duration (days)						
Mean (±SD)			861.09	875.83	-	
Median (IQR)			524.00	(201-1240)	-	
Comorbidities (n, %)						
Cardiovascular diseases	146	9.49	75	9.75	0.01	0.8414
Cerebral vascular diseases	213	13.85	99	12.87	0.03	0.5185
Diabetes	722	46.94	343	44.6	0.05	0.2877
Fatty liver/ Obesity	105	6.83	33	4.29	0.11	0.0155
Medications use (n, %)						
Interferons or nucleosides	278	18.08	179	23.28	0.13	0.0031
Metformin	533	34.66	261	33.94	0.02	0.7332
NSAID	1049	68.21	624	81.14	0.30	<0.0001
Statin	467	30.36	246	31.99	0.04	0.4258
PPI	999	64.95	542	70.48	0.12	0.0079
H2 blocker	812	52.8	491	63.85	0.23	<0.0001
Laboratory data (median, Q1 to Q3)[c]						
AFP (Log transformation)[c,d]						
Mean (±SD)	1.75	1.02	1.70	1.05	-	0.3103
Median (IQR)	1.60	(1.13-2.22)	1.50	(1.04-2.06)	-	0.0823
ALT ≥70 IU/L	374	24·32	182	23.67	0.02	0.7307
Platelets ≥140x 1000/ul	721	46.88	366	47·59	0.01	0.7456
Total Bilirubin >3.0 mg/dl	153	9.95	90	11·7	0.06	0.1954
INR >1.5	105	6.83	59	7.67	0.03	0.4564
HBsAg (+)	388	25.23	207	26.92	0.04	0.3816
HCV antibody (+)	563	36.61	268	34.85	0.04	0.4077
MELD Score[e] (mean±SD)	10.11	3.18	11.30	6.62		0.0032
MELD Score group (n, %)						0.0895
1	1302	84.66	633	82.31	0.06	
2	113	7.35	77	10.01	0.10	
3	123	8	59	7.67	0.01	
4	0	0	0	0	-	

[a]Cases selected by randomly 1:2 PSM by sex, age, cardiovascular disease, cerebrovascular attack, diabetes mellitus, metformin, statin, serum ALT, total bilirubin, HBsAg, anti-HCV antibody, platelet count, prothrombin time by international normalized ratio (INR).

[b]All statistical tests were 2 tailed and used a type I error rate of 0.05 (p).

[c]The log transformation used to address skewed data of AFP.

[d]Laboratory data of AFP were performed using independent-t Test (mean±SD) and Mann-Whitney Test (median, Q1 to Q3)

[e]MELD score variables were performed using independent-t Test (mean±SD)

[f]The chi-square test was used to compare nominal data (n, %).

[g]AFP: alpha-fetoprotein; ALT: alanine aminotransferase; CI: confidence interval, CVD: cardiovascular disease; CVA: cerebrovascular attack; DM: diabetes mellitus; HBV: HBsAg positive; HCV: anti-HCV antibody positive; HR: hazard ratio; INR: international normalized ratio; NSAID: nonsteroidal anti-inflammatory drug; PPI: proton pumping inhibitor; PSM: propensity score matching; SMD: standardized mean difference.

Supplementary Table S3. Clinical characteristics of cirrhotic patients with and without APA treatment (Study C)[a]

	Propensity matched (1:2)					
**Characteristics**	**Untreated**	**(n = 2982)**	**Treated (n = 1491)**		**SMD**	***p*- Value**[b]
Male (n, %)	1844	61.84	934	62.64	0.02	0.6009
Age (mean±SD)	62.06	13.76	63.93	13.54	0.00	<0.0001
Age >65	1343	45·04	659	44.20	0.02	0.595
Aspirin therapy duration (days)						
Mean (±SD)			887.82	1005.·53		
Median (IQR)			457.00	(152-1260)		
Comorbidities (n, %)						
Cardiovascular diseases	346	11.6	173	11.6	0.00	1.0000
Cerebral vascular diseases	439	14.72	243	16.3	0.04	0.1669
Diabetes	1229	41.21	604	40.51	0.01	0.6517
Fatty liver/ Obesity	104	3.49	79	5.3	0.09	0.0039
Medications use (n, %)						
Interferons or nucleosides	453	15.19	323	21.66	0.17	<0.0001
Metformin	745	24.98	377	25·29	0.01	0.8263
NSAID	1716	57.55	1126	75.52	0.39	<0.0001
Statin	587	19.68	270	18.11	0.04	0.2067
Fibrate	89	2.98	95	6.37	0.16	<0.0001
PPI	1642	55.06	1009	67.67	0.26	<0.0001
H2 blocker	1243	41.68	900	60.36	0.38	<0.0001
**Laboratory data (median, Q1 to Q3)**						
AFP (Log transformation)[c,d]						
Mean (±SD)	1.81	1.09	1.70	1.07	-	0.0071
Median (IQR)	1.59	(1.11-2.20)	1.48	(1.02-2.03)	-	0.0002
ALT ≥ 70 **IU/L**	548	20.33	298	20.40	0.00	0.9570
Platelets ≥ 140x 1000/ul	1055	39.59	663	45.76	0.13	0.0001
Total Bilirubin > 3.0 mg/dl	415	15.88	199	13.88	0.06	0.0900
INR >1.5	360	14.75	156	11.26	0.10	0.0023
HBsAg (+)	641	31.89	317	28.66	0.07	0.0616
HCV antibody (+)	790	34.2	448	34.36	0.00	0.9241
MELD Score (mean±SD)[e]	9·72	6.12	11.26	6.96		<0.0001
MELD Score group (n, %)						0.2910
1	2554	85.65	1256	84.24	0.04	
2	191	6.41	96	6.44	0.00	
3	237	7.95	139	9.32	0.05	
4	0	0	0	0	-	

[a]Cases selected by randomly 1:2 PSM by sex, age, CVD, CVA, DM, metformin, statin.

[b]All statistical tests were 2 tailed and used a type I error rate of 0.05 (p).

[c]The log transformation used to address skewed data of AFP [In(AFP)]

[d]Laboratory data of AFP were performed using independent-t Test (mean±SD) and Mann-Whitney Test (median, Q1 to Q3).

[e]MELD score variables were performed using independent-t Test (mean±SD).

[f]The chi-square test was used to compare nominal data (n, %).

[g]AFP: alpha-fetoprotein; ALT: alanine aminotransferase; CI: confidence interval, CVD: cardiovascular disease; CVA: cerebrovascular attack; DM: diabetes mellitus; HBV: HBsAg positive; HCV: anti-HCV antibody; HR：hazard ratio; INR: international normalized ratio; NSAID: nonsteroidal anti-inflammatory drug; PPI: proton pumping inhibitor; PSM: propensity score matching; SMD: standardized mean difference.

Supplementary Table S4. Clinical characteristics of cirrhotic patients with and without APA treatment (Study D, laboratory data included for PSM)[a]

	Propensity Matched (1:2)					
**Characteristics**	**Untreated (n = 2036)**		**Treated (n = 1018)**		**SMD**	***p* - Value**[b]
Male (n, %)	1329	65.28	662	65.03	0.01	0.8932
Age (mean±SD)	61.24	14.08	63.33	13.63	0.00	0.0001
Age >65	873	42.88	433	42.53	0.01	0.8563
Aspirin therapy duration (days)						
Mean (±SD)			848.52	945.39	-	
Median (IQR)			437.00	(157-1236)	-	
Comorbidities (n, %)						
Cardiovascular diseases	169	8.3	96	9.43	0.04	0.2958
Cerebral vascular diseases	318	15.62	166	16.31	0.02	0.6238
Diabetes	877	43.07	418	41.06	0.04	0.2884
Fatty liver/ Obesity	110	5.4	61	5.99	0.03	0.5042
Medications use (n, %)						
Interferons or nucleosides	351	17.24	270	26.52	0.23	<0.0001
Metformin	512	25.15	241	23.67	0.03	0·3731
NSAID	1344	66.01	802	78.78	0.29	<0.0001
Statin	435	21.37	201	19.74	0.04	0.2984
Fibrate	74	3.63	65	6.39	0.13	0.0006
PPI	1359	66.75	742	72.89	0.13	0.0006
H2 blocker	1015	49.85	666	65.42	0.32	<0.0001
Laboratory data (median, Q1 to Q3)						
AFP (Log transformation)[c,d]						
Mean (±SD)	1.77	1.03	1.65	1.00	-	0.0074
Median (IQR)	1.57	(1.10-2.22)	1.46	(1.03-2.03)	-	0.0007
ALT ≥ 70 **IU/L**	413	20.28	209	20.53	0.01	0.8738
Platelets ≥ 140x 1000/ul	821	40.32	434	42.63	0.05	0.2216
Total Bilirubin > 3.0mg/dl	325	15.96	159	15.62	0.01	0.8063
INR >1.5	258	12.67	132	12.97	0.01	0.8181
HBsAg (+)	515	25.29	283	27.8	0.06	0.1375
HCV antibody (+)	691	33.94	340	33.4	0.01	0.7660
MELD Score (mean±SD)[e]	10·54	6.53	11.86	7.16		0.0008
MELD Score group (n, %)						0.3522
1	1696	83.3	854	83.89	0.02	
2	155	7.61	64	6.29	0.05	
3	185	9.09	100	9.82	0.03	
4	0	0	0	0	-	

[a]Cases selected by randomly 1:2 PSM by sex, age, cardiovascular disease, cerebrovascular attack, diabetes mellitus, metformin, statin, serum ALT, total bilirubin, HBsAg, anti-HCV antibody, platelet count, prothrombin time by international normalized ratio.

[b]All statistical tests were 2 tailed and used a type I error rate of 0.05 (p).

[C]The log transformation used to address skewed data of AFP.

[d]Laboratory data of AFP were performed using independent-t Test (mean±SD) and Mann-Whitney Test (median, Q1 to Q3)MELD score variables were performed using independent-t Test (mean±SD).

[e]MELD score variables were performed using independent-t Test (mean±SD).

[f]The chi-square test was used to compare nominal data (n, %).

[g]AFP: alpha-fetoprotein; ALT: alanine aminotransferase; CI: confidence interval, CVD: cardiovascular disease; CVA: cerebrovascular attack; DM: diabetes mellitus; HBV: HBsAg positive; HCV: anti-HCV antibody; HR: hazard ratio; INR: international normalized ratio; NSAID: nonsteroidal anti-inflammatory drug; PPI: proton pumping inhibitor; PSM: propensity score matching; SMD: standardized mean difference.

Supplementary Table S5. The incidences of hepatocellular carcinoma at three and five years of follow-up (Study A and C)

		Case no.	HCC	Incidence (%)	Mean Follow-up (y)	Total Follow-up (y)	Incidence per 100 Person-Year	Events/Cases
Aspirin (at 3 y)	Treated	1135	47	4.14%	2.78	3154.10	1.49	47/1135
	Untreated	2270	181	7.97%	2.34	5304.56	3.41	181/2270
Aspirin (at 5 y)	Treated	1135	82	7.22%	3.37	3822.95	2.14	82/1135
	Untreated	2270	248	10.93%	4.32	9794.78	2.53	248/2270
APAs (at 3 y)	Treated	1491	93	6.24%	2.50	3726.99	2.49	93/1491
	Untreated	2982	265	8.89%	2.25	6708.66	3.95	265/2982
APAs (at 5 y)	Treated	1491	138	9.26%	3.19	4761.65	2.90	138/1491
	Untreated	2982	356	11.94%	3.723	11102.84	3.21	356/2982

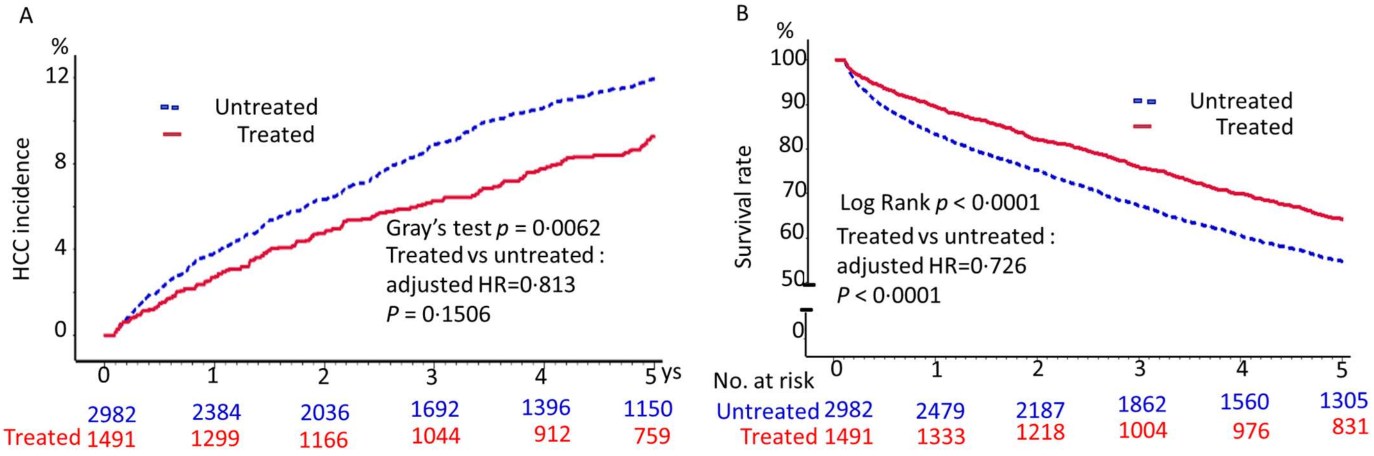

Figure S1. Cumulative incidence of HCC **(A)** and survival **(B)** among APA users and nonusers. Non-aspirin antiplatelet agent (APA) use was defined as a refilled prescription for 84 or more consecutive days after the index date; nonuse was defined as no use. For those APA use fewer than 84 consecutive days were excluded. (A) P value was determined using Gray's test. (B) P value, the log-rank test.

Supplementary Table S6. Effect of APAs or aspirin use on risk of incident hepatocellular carcinoma (Study B and D)[a,b]

Clinical Outcome and Treatment Group	Number with Event/ Total No.	Incidence Rate (per 100 Person-Year)	Univariate Cox Model				Multivariable Cox Model			
			Crude HR	(95% CI)		*p* -Value	Adjusted HR	(95% CI)		*p* -Value
3-years outcomes										
**Incident HCC**										
Aspirin use	28/769	1.30	0.47	0.31	0.71	0.0004	0.49	0.32	0.76	0.0012
APAs	56/1018	2.19	0.65	0.48	0.88	0.0049	0.81	0.58	1.14	0.2249
5-years outcomes										
**Incident HCC**										
Aspirin use	48/769	1.76	0.62	0.45	0.85	0.0034	0.62	0.45	0.88	0.007
APAs	77/1018	2.29	0.68	0.52	0.88	0.0031	0.86	0.64	1.14	0.2935

[a]PSM variables： sex, age, cardiovascular disease, cerebrovascular attack, diabetes mellitus, metformin, statin, serum ALT, total bilirubin, HBsAg, anti-HCV antibody, platelet count, prothrombin time by international normalized ratio.

[b]Using the Fine and Gray method to take causes mortality into account all as a competing risk of incident HCC.

[c]CI: confidence interval; HR: hazard ratio; PSM: propensity score matching. APAs: non-aspirin antiplatelet agents.

Supplementary Table S7. Effect of daily non-aspirin antiplatelet agent use on risk of incident gastrointestinal bleeding[a,b].

Clinical outcome and Treatment Group	Multivariable Cox Model			
	Adjusted HR	(95% CI)		*p* - Value
**Patients without previous GI bleeding**				
3-year outcome	0.80	0.61	1.05	0.1132
5-year outcomes	0.81	0.61	1.06	0.1206
**Patients with previous GI bleeding**				
3-year outcome	0.71	0.53	0.94	0.0162
5-year outcomes	0.66	0.51	0.84	0.001

[a]PSM by sex, age, cardiovascular disease, cerebrovascular attack, diabetes mellitus, metformin, statin, serum ALT, total bilirubin, HBsAg, anti-HCV antibody, platelet count, prothrombin time by international normalized ratio.

[b] Using the Fine and Gray method to take into account all causes mortality as a competing risk of Incident GI bleeding

[c] AFP: alpha-fetoprotein; ALT: alanine aminotransferase; CI: confidence interval, CVD: cardiovascular disease; CVA: cerebrovascular attack; DM: diabetes mellitus; HBV: HBsAg positive; HCV: anti-HCV antibody positive; HR: hazard ratio; INR: international normalized ratio; PSM: propensity score matching.
